# 3D bio-printed hydrogel inks promoting lung cancer cell growth in a lab-on-chip culturing platform

**DOI:** 10.1007/s00604-023-05931-8

**Published:** 2023-08-12

**Authors:** Agnieszka Krakos, Adrianna Cieślak, Eliza Hartel, Magdalena Beata Łabowska, Julita Kulbacka, Jerzy Detyna

**Affiliations:** 1 Department of Microsystems, Faculty of Electronics, Photonics and Microsystems, Wroclaw University of Science and Technology, Janiszewskiego 11/17, 50-372 Wroclaw, Poland; 2 Department of Mechanics, Materials and Biomedical Engineering, Faculty of Mechanical Engineering, Wroclaw University of Science and Technology, Smoluchowskiego 25, 50-371 Wroclaw, Poland; 3 Department of Molecular and Cellular Biology, Wroclaw Medical University, Borowska 211A, 50-556 Wroclaw, Poland; 4 Department of Immunology, State Research Institute Centre for Innovative Medicine, Vilnius, Lithuania

**Keywords:** Lab-on-chip, LOC, 3D bio-print, Hydrogel, Cell culture, Biomaterials

## Abstract

The results of a lab-on-chip (LOC) platform fabrication equipped with a hydrogel matrix is reported. A 3D printing technique was used to provide a hybrid, “sandwiched” type structure, including two microfluidic substrates of different origins. Special attention was paid to achieving uniformly bio-printed microfluidic hydrogel layers of a unique composition. Six different hydrogel inks were proposed containing sodium alginate, agar, chitosan, gelatin, methylcellulose, deionized water, or 0.9% NaCl, varying in proportions. All of them exhibited appropriate mechanical properties showing, e.g., the value of elasticity modulus as similar to that of biological tissues, such as skin. Utilizing our biocompatible, entirely 3D bio-printed structure, for the first time, a multi-drug-resistant lung cancer cell line (H69AR) was cultured on-chip. Biological validation of the device was performed qualitatively and quantitatively utilizing LIVE/DEAD assays and Presto blue staining. Although all bio-inks exhibited acceptable cell viability, the best results were obtained for the hydrogel composition including 3% sodium alginate + 7% gelatin + 90% NaCl (0.9%), reaching approximately 127.2% after 24 h and 105.4% after 48 h compared to the control group (100%). Further research in this area will focus on the microfluidic culture of the chosen cancer cell line (H69AR) and the development of novel drug delivery strategies towards appropriate in vivo models for chemotherapy and polychemotherapy treatment.

**Graphical abstract:**

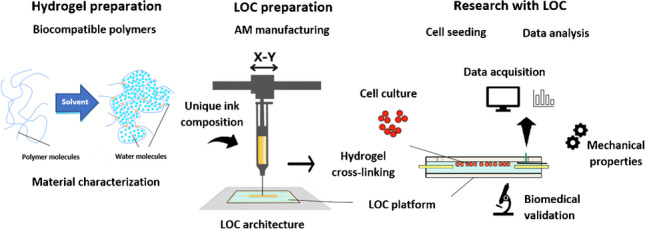

## Introduction

Microfluidics and microfluidic-related lab-on-chip (LOC) techniques have their origin in the silicon and silicon-glass microengineering dating back to the early 1960s, when the first microelectro-mechanical system (MEMS)-based sensors were fabricated [1]. Based on these approaches, the evolution of the so-called micro total analysis system (µTAS) instrumentation, usually taking the form of LOC microfluidic devices, has appeared in the literature.

**Fig. 1:**
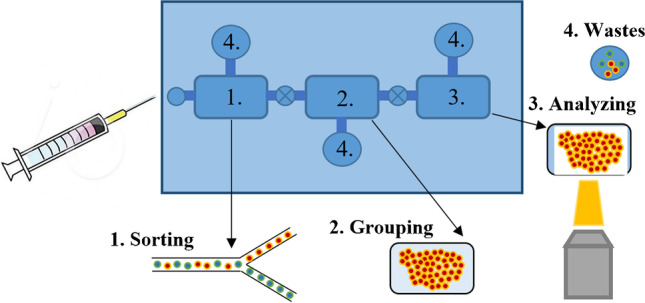
The concept of µTAS instrumentation, based on [2]

The µTAS concept was firstly presented at the International Conference on Miniaturized Systems for Chemistry and Life Science (µTAS 1994) by Prof. P. Bergveld [2]. His idea of fully integrated microfluidic system, equipped with microvalves, microdosers, microchannels, microseparators, etc. (Fig. 1), ensuring comprehensive sample analysis within a single LOC, has notably changed the miniaturization aspects and indicates possible benefits with science worldwide.

LOCs are usually fabricated out of glass, silicon, ceramics, and polymers, e.g., polystyrene (PS), cyclic olefin copolymer (COC), or polydimethylsiloxane (PDMS), in a form of hybrid structures [3–7]. Typically, standard micromachining techniques are used herein to ensure substrate patterning, i.e., photolithography, chemical etching, bonding, soft lithography, and molding, but nowadays, a technique of 3D printing is also gaining in popularity [8–10].

At recent times, different 3D printing methods can be distinguished, e.g., process fused deposition (FDM), digital light process (DLP), selective laser sintering (SLS), or two-photon polymerization [11–13]. Depending on the technique applied, diverse materials and facilities are employed, which often determines the final product potential. 3D printing technique is more and more being used for the fabrication of very small objects and forming of complex microfluidic structures at the high level of detail, also for *life science* field [14–18].

Typically, LOCs are used for biomedical investigation covering cell cultivation, drug screening, DNA analysis, etc. [19–21]. Nevertheless, the basic problems with LOCs, which notably inhibit their global popularization in clinical research, are biocompatibility and possible, often indefinable time degradation [22, 23]. On that basis, a strong trend towards novel microfluidic substrates and techniques is being developed, coming into the scope of hydrogel matrices.

Hydrogels of unique composition can imitate cells’ natural in vivo habitat which is the best way to provide physiological growth for cell cultures in vitro. Moreover, the specific character of the hydrogel ensures the development of cell cultures in a form of three-dimensional objects, encapsulated within the hydrogel bulk which additionally improves the cultivation process [14]. According to Sun et al. [24], hydrogel-based LOCs may gradually replace standard microfluidic materials and become the basic ones shortly.

Generally, hydrogels can be defined as three-dimensionally cross-linked chains of a hydrophilic polymeric material. Hydrogels are formed by the reaction of one or more monomers or through associative bonds — physical (hydrogen bonds, electrostatic interactions, van der Waals forces between chains) or chemical (covalent bonds). Hydrogels are able to retain a large volume of liquid between polymer chains. This ability results from the presence of hydrophilic functional groups attached to the polymer backbone, i.e., -OH, -COO^–^, -COOH, > C = O, -CONH_2_, > CHNH_2_, -NH_2_, and -SO_3_H [25–28]. The hydrogel’s polymer networks have a water absorption capacity of up to a thousand times their dry weight, with an arbitrarily lower limit of 10% [29, 30].

Hydrogels are often used in additive manufacturing technology applications due to their easy processability. This enables the fabrication of three-dimensional structures by successively injecting material layer by layer. The additive manufacturing technology provides reproducible, complex shapes without material loss and often without the need for additional tooling [31–34]. The hydrogel materials used in 3D bioprinting technology are known as inks. Over nearly 40 years, additive manufacturing technology has continuously evolved, allowing the processing of more and more complex materials. Currently, this technology creates the possibility of using cells inside inks. However, the process parameters need to be optimized due to, for example, the possibility of damaging the cells with excessive pressure [35, 36]. 3D bioprinting, as one of the subgroups of material jetting, is the most commonly used method for processing hydrogels mixed with living cells or proteins, to create geometrically complex scaffolds for cell cultures, as well as tissue constructs, or spheroids [37–39]. In the case of exclusively LOC solutions, these are typically hydrogel-based cell analysis platforms for migration, drug resistivity, and cultivation studies [40–43].

Although the process of 3D printing is an emerging field now, several issues still need to be solved to ensure widespread adoption of this technique for hydrogel-based LOC fabrication. First and foremost, the critical issue encompasses the selection of the most appropriate material, which apart from the suitable mechanical and rheological properties, will guarantee the physiological growth of the cells. According to the paper [10], gelatin-based hydrogels which are considered the most popular recently are not the best choice for microfluidics, due to poor mechanical properties, high viscosity, and sensitivity to enzymatic degradation and some new approaches have to be proposed to assure printing of small-scale geometries, i.e., microchannels/microchambers/microvias and thus, fulfill the user-defined structure complexity. Herein, repeatability aspect of the process also cannot be forgotten, as it determines the widespread of the 3D printed hydrogel LOCs, towards future commercialization of these models, as more physiologically relevant in comparison to standard PDMS LOCs [44]. As it is not trivial, many LOC-based studies still utilize hydrogel casting instead of 3D printing, to obtain the substrate of precise shape and size [45, 46].

With a view to the current literature [10, 47], it has to be said that the development of hydrogel LOC platforms for cell cultivation research is still at the level of basic research. Neither optimal hydrogel composition nor 3D printing parameters have been optimized to date to fulfill the demands of cell culture management on-chip. Even if the chosen material is well printable, mechanically durable, exhibits moderate time-dependent stability, and can be applied for the investigation of the selected cell line, there is no guarantee it will be appropriate for the other microbial object [48]. The recent literature positions [49, 50] indicate that multi-material approach may solve this problem and provide more universal environment for cells culture; however, it is still challenging due to the necessity of new 3D printer tools. Some ready-to-use hydrogel mixed starting compounds are recently available [41, 51], but can be used solely with the dedicated printer models and are considered as quite expensive.

Based on all of this, further research on the hydrogel composition, 3D printing applicability, and biomedical utility is needed to define the baselines and overcome any technological barriers for development of reliable, 3D culture model LOCs. As the oncological purposes, e.g., chemotherapy and investigation of cell drug resistivity with novel drug-delivery strategies, are the most commonly discussed with LOCs nowadays [52], the new knowledge on cancer cell line behavior in hydrogel-based microenvironments imitating in vivo conditions is crucial.

In this paper, for the first-time, lung cancer cell line (H69AR) was chosen to be studied on-chip utilizing 3D printed hydrogel layers of unique composition. Research described in this paper was undertaken to fill the gap concerning fabrication of microfluidic solutions which are compatible with hydrogel bio-printing technique and simultaneously, ensure cell culturing of highly sensitive and drug-resistant lung cancer cell line [53]. Commercially available bio-printer was used to provide the hydrogel structure of defined geometry, made of specially prepared sodium alginate mixtures. Thus, new knowledge on hydrogel multi-material performance, bio-print technology, and biological applicability is shown under the frames of this work.

## Materials and methods

In the paper, a LOC fabricated from scratch with the use of biocompatible materials is shown. In this solution, a commercially available multi-jet 3D printed substrates have been used as a “base” and a chip “cover,” and self-developed hydrogel of the unique composition as an intermediate, 3D printed layer. Details on the LOC design, technology, structure stability, and preliminary biological validation are shown in the following sections of this article.

### Design of LOC

**Fig. 2:**
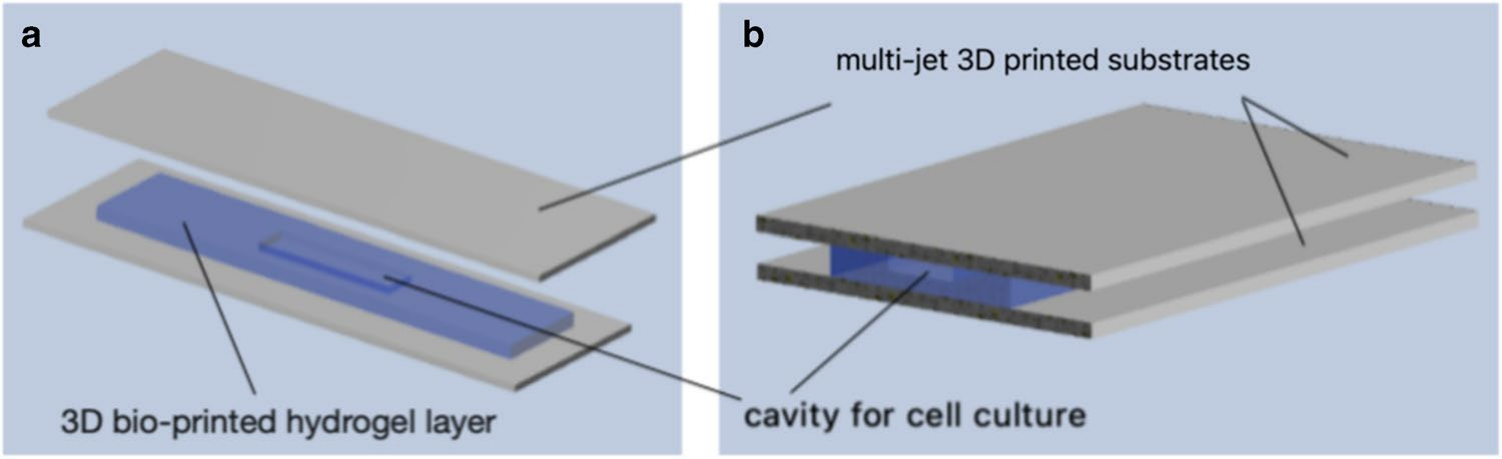
General scheme of the LOC: (**a**) exploded schematic view, (**b**) assembled structure

A simple design of the LOC was proposed herein to provide technological proof-of-concept. The basic assumption was to create a 1-mm-deep hydrogel cavity of the 20 × 5-mm dimensions and rectangular shape in the center of the hydrogel matrix. Such a solution ensures potential cell inoculation for cell cultivation experiments. The overall size of the 3D bio-printed hydrogel matrix was smaller than the multi-jet 3D printed substrates to allow for convenient structure cross-linking with CaCl_2_. The hydrogel’s dimensions were intended to be 64 × 14 × 2 mm. The sandwich-type LOC is shown in Fig. 2.

### Fabrication of LOC substrates with a hydrogel layer

As mentioned earlier in this paper, biocompatible 3D printed substrates have been fabricated as a LOC “base” and a “cover” for the hydrogel matrix placed between.

**Fig. 3:**
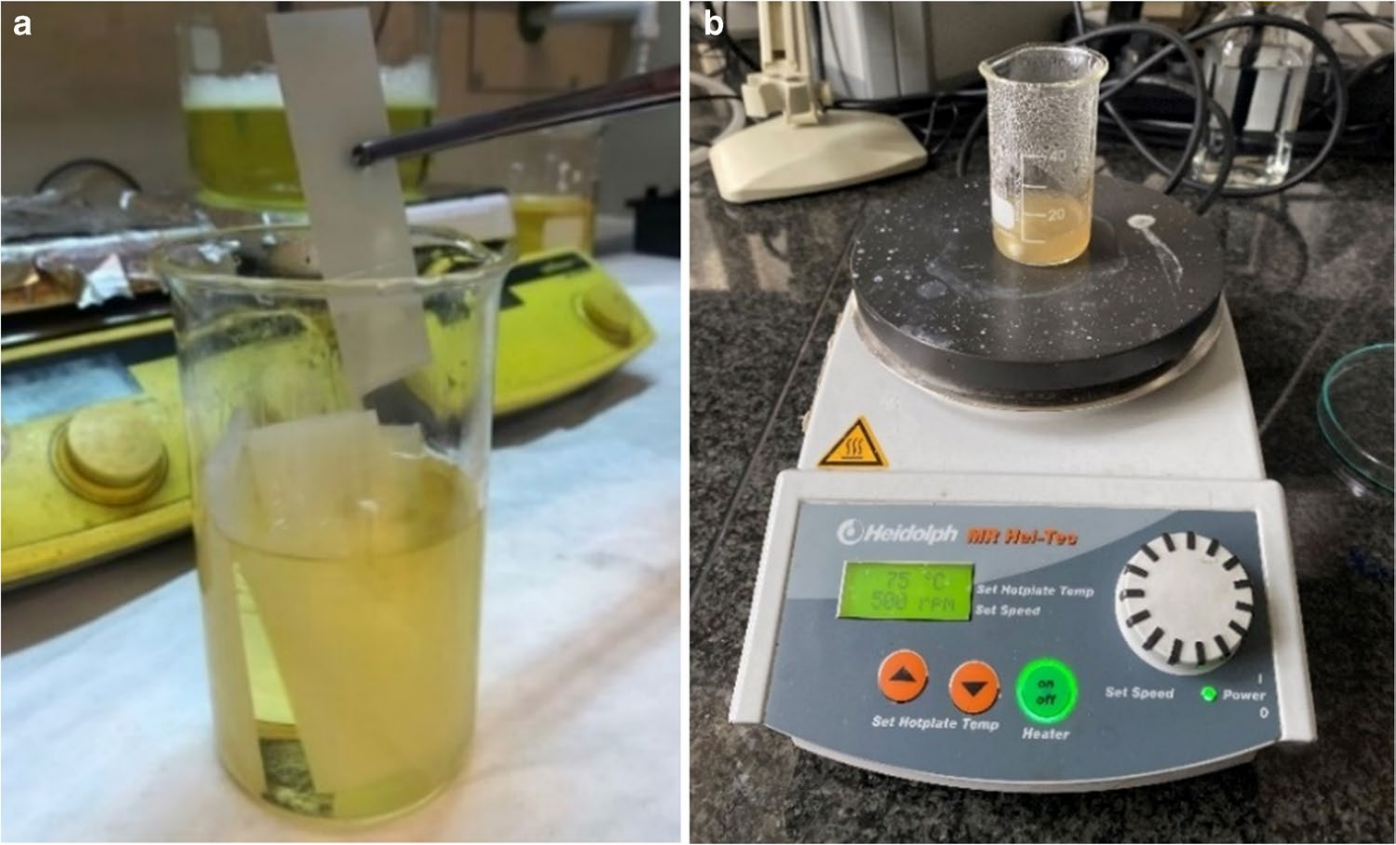
Preparation of the LOC substrates: (**a**) multi-jet 3D printed substrates prior to heating in oil-bath, (**b**) preparation of polymer solution for hydrogel matrices

For this purpose, multi-jet 3D printer (model: Projet 3500 HD Max, 3D Systems, USA) was used with photocurable resin – VisiJet M3 Crystal (3D Systems, USA) and support material (VisiJet S300, 3D Systems, USA) to achieve rectangular substrates of 76 × 26 × 1.1 mm^3^. This printer model prints thin layers of UV-curable liquid resin (VisiJet M3 Crystal, 3D Systems, USA) onto a platform, using wax (VisiJet S300, 3D Systems, USA) to create supports that brace the part during production. UV lamps cure each layer of the resin, and the process continues layer by layer until the part is complete. The post-processing of the structures was done by heating in an oil bath at 65 °C for the desired time to remove the support material, wax (Fig. 3a). Then, washing with a detergent at an elevated temperature (65 °C) was applied, followed by cleaning with isopropyl alcohol (IPA), according to the procedure described elsewhere [8].

To create hydrogel matrices, six polymer solutions of various compositions were prepared. Three of which were manufactured using deionized (DI) water and the other three were created using a saline solution (Fresenius Kabi, Poland) containing 0.9% NaCl solution. Polymer solution compositions were created using sodium alginate (Sigma-Aldrich, USA) with viscosity ranging from 5.0 to 40.0 cps (*c* = 1% in water at 25 °C), agar (Agnex, Poland) with viscosity ranging from 10 to 100 cps (*c* = 1.5% in water at 60 °C), chitosan (Sigma-Aldrich, Iceland) with viscosity ranging from 20 to 300 cps (*c* = 1% in 1% acetic acid), gelatin (Warchem, Poland), and methylcellulose (Sigma-Aldrich, China) with viscosity ranging from 12 to 18 cps (*c* = 2% in water at 20 °C). All polymers were intended for cell culture purposes. Table 1 includes a list of all polymer solution formulations and their concentrations. The procedure for hydrogel manufacturing is described below.

**Table 1:** Composition of the hydrogel inks

Sample symbol	Composition of polymer inks
Hydrogels based on DI water	
H1	Sodium alginate (4%) + chitosan (4%)
H2	Sodium alginate (4%) + gelatin (4%)
H3	Sodium alginate (4%) + agar (2%) + methylcellulose (2%)
Hydrogels based on saline solution	
H4	Sodium alginate (3%) + gelatin (7%)
H5	Sodium alginate (4%) + chitosan (4%)
H6	Sodium alginate (4%) + agar (2%) + methylcellulose (2%)

The hydrogel preparation procedure involved mixing the polymeric powders with DI water (samples H1, H2, H3) or saline solution (samples H4, H5, H6) in appropriate proportions using a magnetic stirrer (500 rpm) at 60 °C for 90 min (Fig. 3b). Through this initial step, a polymer solution was created and then used as ink in the 3D bioprinter.

The prepared ink (polymer solution) was applied on a LOC substrate using a Cellink BIO X 3D printer.^1^ After fabrication of the polymeric structures using the aforementioned 3D bioprinter, calcium chloride (CaCl_2_) (Avantor Performance Materials Poland, Poland) was used to cross-linking the polymeric solution. The cross-linking agent’s concentration (calcium chloride solution) was 0.1 M and the cross-linking time was 10 min. Consequently, hydrogel structures were formed, ready to be loaded with cells.

### Cell line-based research investigations

An epithelial lung cancer cell line H69AR (ATCC®, USA) was used as a model for the biological validation of the LOCs. H69AR cells represent Adriamycin resistance, a drug used in lung cancer therapy [54]. This cell line is often used as a model for 3D cultures and spheroids [55–57] and in particular for drug resistance overcoming in terms of the improving efficacy of chemotherapeutics [54, 58, 59]. Despite so many advantages, this cell line has not been studied in the context of lab-on-chip using hydrogel substrates. In our research, we attempted to use H69AR cells in the context of creating 3D cultures and their potential application in studying drug resistance and anticancer therapies.

### Biological validation of the LOC

Working in a microbiology laboratory involves maintaining cleanliness and sterility. During the research with biological cells, single-use gloves and lab coats were used. The disinfection of equipment or hands was performed by washing with a 70% alcohol solution. The laminar flow cabinet (Thermo Scientific MSC Advantage, Argenta Lab, Poland) was turned on 3 min before starting work. Only sterilized disposable or reusable laboratory instruments were used during the experiments. Proper and safe disposal of biological and chemical wastes is also important. After the work was completed, the laminar flow cabinet was washed with an alcohol solution and the built-in UV lamp, which performs sterilizing functions, was turned on. Cell culture conditions are the essence of obtaining reliable and credible research results. Biological experiments were conducted at the Faculty of Pharmacy of the Wroclaw Medical University in the Department of Molecular and Cellular Biology. Cell culture was carried out in sterile culture bottles in a monolayer. The cells were mixed with RPMI (RPMI-1640, Biological Industries Israel Belt Haemek Ltd, Israel) culture fluid, which provides the required nutrients (amino acids, vitamins, glucose, etc.) and is responsible for buffering the environment. RPMI was supplemented with a factor that stimulates cell proliferation, namely 5% fetal bovine serum — FBS. The cells immersed in a culture buffer, after trypsinization, were applied from the macroscale bottle to the cavity of a hydrogel matrix. During the cultivation time, the LOCs were placed in the incubator (Standard BD Avantgarde. Line, Binder, Poland) with a temperature setting of 37 °C and an atmosphere of 5% CO_2_. Cell cultures were observed at 24- and 48-h intervals. Observation of cells in the LOC platform was carried out using a Leica DMi1 inverted microscope (Leica Microsystems, Germany), which was used to verify the presence of cells both in the cavity and throughout the hydrogel at different depths. Researchers investigated cell viability with two different tests to present quantitative and qualitative results of cell biological activity. The LIVE/DEAD assay was carried out by measuring fluorescence after cell incubation (30 min) in the presence of each hydrogel in Presto Blue reagent (Thermo Fischer Scientific, Oregon) — quantitative evaluation. The results are shown in the Table 3 and in Fig. 5. In turn, a qualitative assessment shown in Fig. 8 was performed using trypan blue dye (0.5%) (Biological Industries, Israel) and microscopy observation. Each test was performed 4 times for all hydrogels. In parallel, the reference cell culture was a solution of cancer cells in a culture medium applied to small Petri dishes. The control group provided a reference (cell viability assumed to be 100%) to the results obtained from the research groups.

### Evaluation of hydrogel stability

To check the stability of the prepared structures, the hydrogel samples were tested towards compression strength without and after exposure to standard incubation conditions utilizing MultiTest-i-1 instrument (Mecmesin, UK) equipped with an ILC-S strain gauge sensor with a measuring range of 100 N. The head enables force measurement with an accuracy of 0.01 N and displacement measurement with precision of 0.0025 mm. For this purpose, the samples were placed in an Incu-Line incubator with natural convection (temperature control: 0.1 °C, temperature range: 5–70 °C) providing *T* = 37 °C and flooded with saline solution. The incubation time was 24 h and 48 h, respectively, to pre-simulate the environmental conditions of the cells, during their culture. The control group consists of hydrogel samples compressed straight after cross-linking (without incubation). Each case was repeated 6 times, resulting in a total number of 108 tested samples.

### Statistical analysis procedure

Statistical analyses were performed with Statistica package (version 13, TIBCO Software Inc., USA). The significance level was assumed at *p* < 0.05. In each case, the nature of the normal distribution had to be verified. If the distribution was normal (Shapiro-Wilk test, *p* ≥ 0.05), an assessment of homogeneity of variance was made (Brown-Forsythe test, *p* ≥ 0.05). When compared multiple independent groups, ANOVA tests were carried out. The distribution was other than normal, so non-parametric tests were performed. One example of ANOVA tests is the Kruskal-Wallis post hoc test, typically employed in the case of multiple independent groups.

One way to visualize the results is a “box-and-whisker” chart, which facilitated the interpretation of the regularities present. If a normal distribution is present, the following is indicated: the mean and two standard deviations. Otherwise, median, quartiles, and the spread, i.e., the minimum and maximum values are calculated.

## Results

### LOC fabrication utilizing 3D bio-printing with hydrogel inks

It was noticed that the key parameters for the polymer solution printing process are as follows: the temperature of the extruded mixture (set by heating the printhead), the speed at which the head with the syringe moves along the given trajectory, the pressure at which the hydrogel is extruded, the temperature of the working table and the head heating the syringe (Table 2).

**Table 2:** Parameters of the bioprinting process

Parameter	Hydrogel composition	
	Sodium alginate, agar, methylcellulose	Sodium alginate, gum Arabic, methylcellulose
Polymeric solution temperature (°C)	28	28
Printhead speed (mm/s)	15	13
Extrusion pressure (kPa)	10	12
Table temperature (°C)	27	27
Head temperature (°C)	60	60

**Fig. 4:**
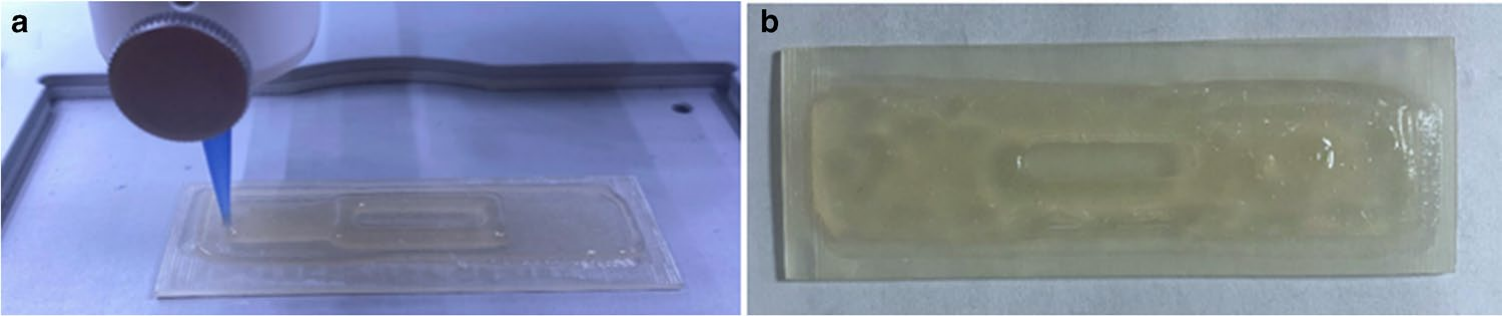
Structure of LOC: (**a**) manufacturing process of hydrogel layer by 3D bioprinter, (**b**) final cross-linked structure before cell culture placement

As the parameters of the bio-printing process were fitted based on the hydrogel composition, deposited layers were ultimately in good relation to the model geometries (Fig. 4).

The ability to heat the print head and the syringe placed in it, and therefore also the heating of the ink prevents polymerization of the material before the printing process. When developing the composition of the filament, particular attention was taken to ensure that the components were selected in such proportions that the hydrogel was easily formable, i.e., the properties of its ingredients in appropriate proportions allowed for the mirroring of the models’ geometry during printing. Otherwise, a polymer solution that has too low density could spill over the printout. If the material was too viscous, it could easily solidify in the syringe or the printing process could be limited because the maximum extrusion pressure is 200 kPa.

Crucially, the hydrogel-forming stage began by creating permanent bonds using a cross-linking agent applied to the printed polymer solution.

### Biological validation of the LOC

**Fig. 5:**
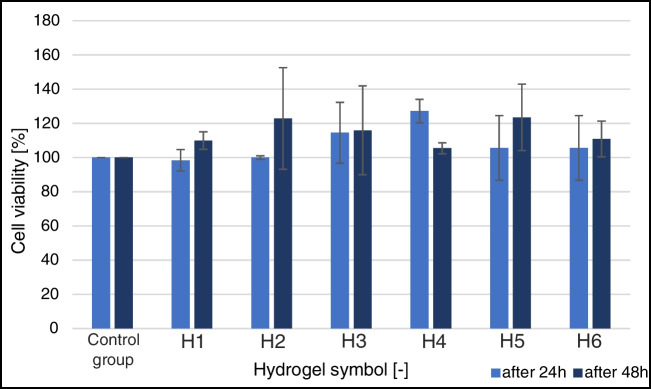
Results of the H69AR lung cancer cell viability test in hydrogels after 24 and 48 h (*N* = 4). The control group (cells in culture medium) was taken as 100%. Results are presented as mean ± standard deviation

The results of the cytotoxicity tests of the prepared hydrogels demonstrated their biocompatibility with cells. The survival rate of H69AR lung cancer cells on different hydrogel formulations based on Presto Blue staining is shown in Table 3 and Fig. 5. Viability for the control group was set to 100%.

**Table 3:** The viability test of H69AR lung cancer cells in hydrogel after 24 and 48 h (*N* = 4)*

Type of hydrogel substrate in LOC		Viability validation (%)	
Symbol	Hydrogel composition	24 h	48 h
Control group	(Without hydrogel)	100	100
H1	Sodium alginate (4%) + chitosan (4%) + DI water	98.4 ± 6.3	109.9 ± 5.1
H2	Sodium alginate (4%) + gelatin (4%) + DI water	100.0 ± 1.0	122.9 ± 29.7
H3	Sodium alginate (4%) + agar (2%) + methylcellulose (2%) + DI water	114.5 ± 17.8	115.9 ± 26.0
H4	Sodium alginate (3%) + gelatin (7%) + NaCl	127.2 ± 6.9	105.4 ± 3.2
H5	Sodium alginate (4%) + chitosan (4%) + NaCl	105.6 ± 18.9	123.5 ± 19.4
H6	Sodium alginate (4%) + agar (2%) + methylcellulose (2%) + NaCl	105.6 ± 18.9	110.9 ± 10.5

As indicated in Table 3, cell development and proliferation are promoted by all the manufactured hydrogels in LOCs. After 24 and 48 h, the cell survival rate of the hydrogels with the following compositions: 3% sodium alginate + 7% gelatin + NaCl solution; 4% sodium alginate + 4% gelatin + DI water; and 4% sodium alginate + 4% chitosan + DI water varied statistically significantly. The combination of 3% sodium alginate + 7% gelatin + NaCl solution (H4) results in a most favorable hydrogel for cell development after 24 h. In meantime, after 48 h, the highest viability results were received for hydrogels H2 and H5. The lower percentage of viability observed for hydrogel based on 4% sodium alginate + 4% chitosan + DI water (especially after 24 h) might be a result of the hydrogel’s stiffness caused by the improper quantity of chitosan. Excessive stiffness of the hydrogel disrupts the morphology of the cells, which can lead to cell death [60]. The other aspect is the acidification of the environment by chitosan which is not suitable for proper cell growth [61]. However, the question of the appropriate ratio of hydrogel based on chitosan remains to be further investigated.

**Fig. 6:**
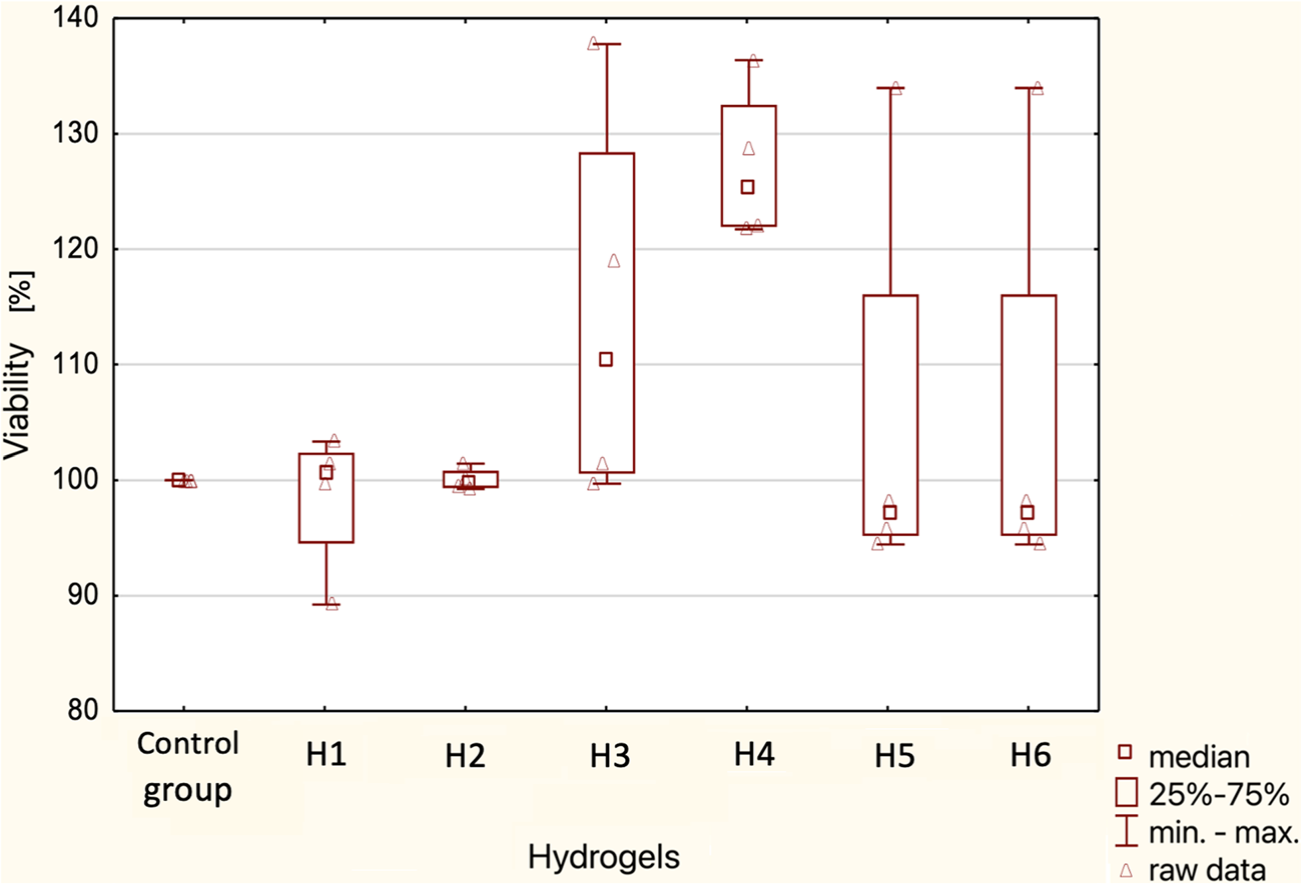
Summary “box-and-whisker” plot for cell viability in the tested hydrogels — test after 24 h. Median characteristics (*N* = 4)

**Fig. 7:**
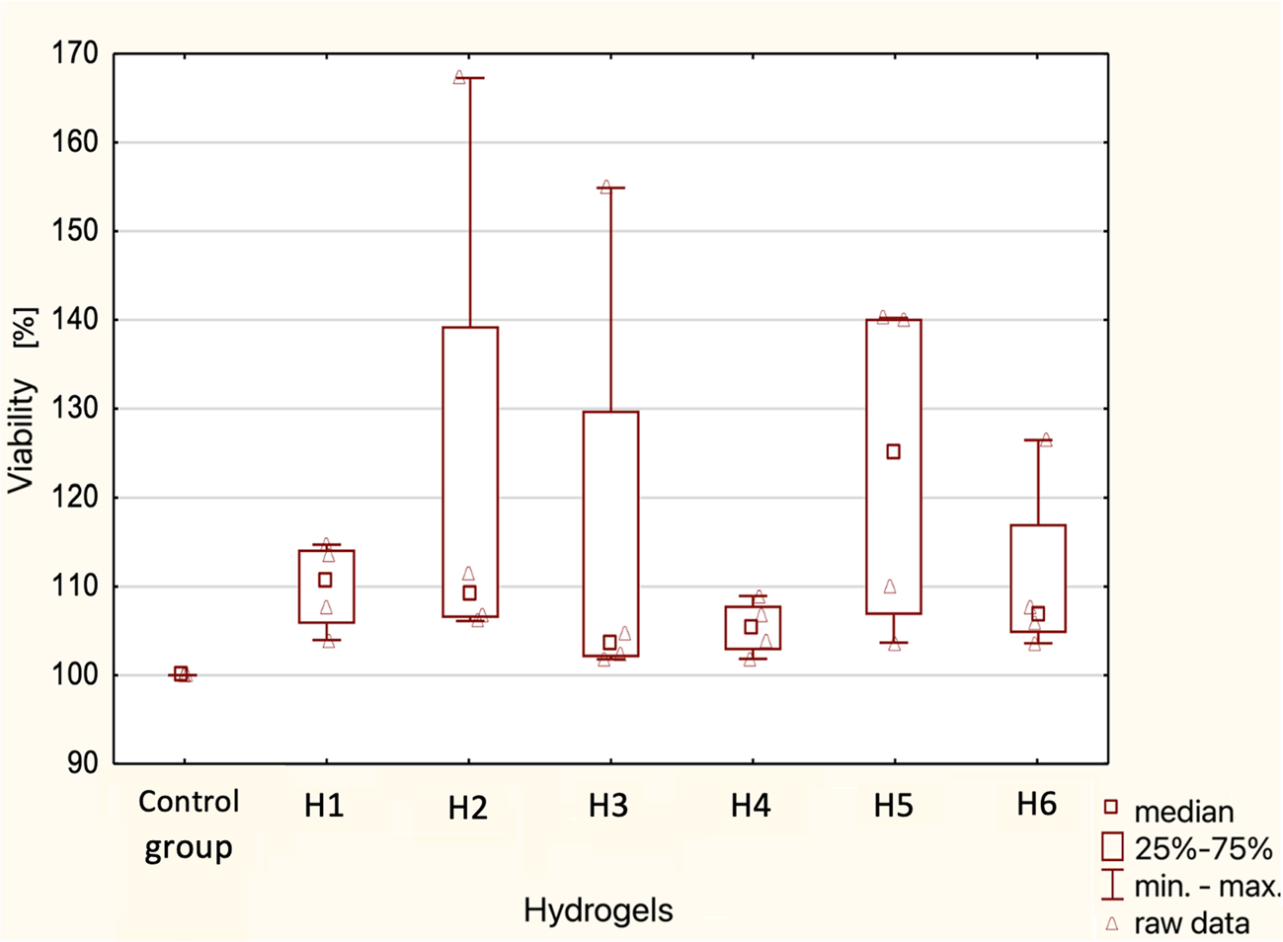
Summary “box-and-whisker” plot for cell viability in the tested hydrogels — test after 48 h. Median characteristics (*N* = 4)

A statistical analysis of the measured viability of lung cancer cells in different hydrogels after 24 h and 48 h was performed. A “box-and-whisker” plot, in which the median, first, and third quartiles, as well as the maximum and minimum values of tumor cell viability, are indicated, is shown in Figs. 6 and 7.

Due to the lack of normal distribution of results, non-parametric tests were carried out - Kruskal-Wallis test. The significance level was assumed at *p* < 0.05. According to the results of the analysis, there are no statistically significant differences between cell viability (%) in different hydrogels after 24 h and 48 h.

**Fig. 8:**
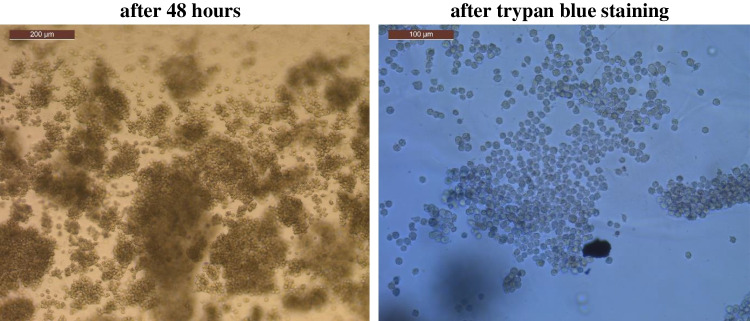
H69AR lung cancer cell culture morphology on saline solution-based hydrogel (sodium alginate (3%) + gelatin (7%)) after 48 h and trypan blue staining

As mentioned earlier, when comparing the results of the cell survival test using water or salt solution as the hydrogel solvent basis, differences between them could be detected. Compared to hydrogels created with DI water, those made with NaCl exhibited a higher survival rate after 48 h. An example of cell colonies cultured utilizing hydrogel layer based on saline solution, sodium alginate (3%) and gelatin (7%), is shown in Fig. 8.

Compared to studies described in research institutions around the world, the results given in this paper are promising. For instance, the viability of cells cultured utilizing hydrogel LOC platforms was verified by Knowlton S. and co-workers [14]. The study was conducted on a methacrylate gelatin-based hydrogel. Comparing the survival of cells cultivated on LOC to a control sample, researchers faced a substantial difference. The viability rate of cell culture on LOC was solely 55% after 24 h. Better results were lastly presented by Mohamed et al. studying cell viability in gelatin methacrylate (GelMA) microgel and indicated survival rate of cells to 80–90% after 5 days of the experiment [62]. This shows that depending on the cell type, the material choice should also be customized.

### Evaluation of hydrogel stability

**Fig. 9:**
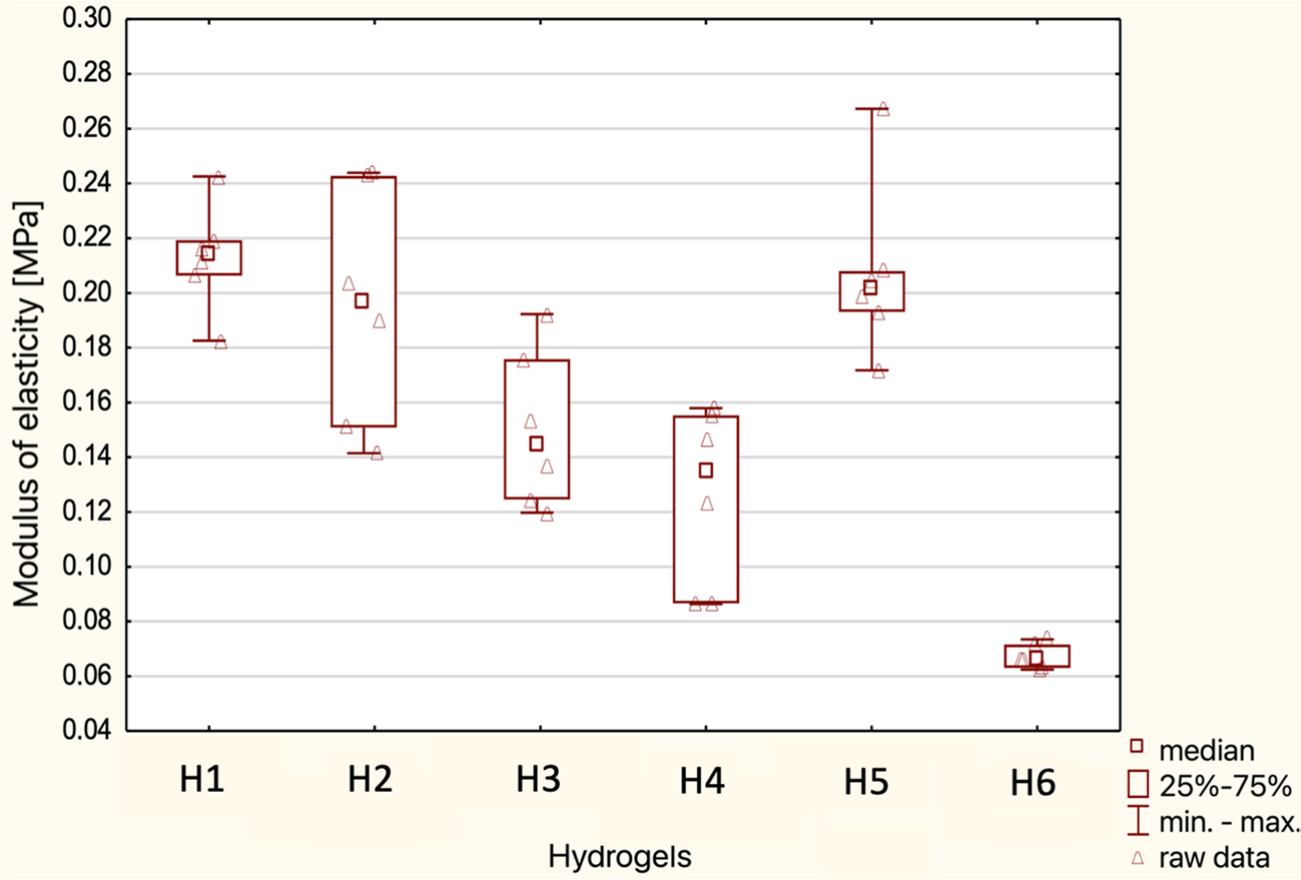
Summary “box-and-whisker” plot for individual hydrogels for elastic modulus measurements after 48 h. Median characteristics (*N* = 6)

Studies of the modulus of elasticity of the prepared hydrogels allowed to assess their degradability under incubation conditions, including the long-term usability and appropriate durability of the structure for cell culture. Based on compression tests of hydrogel samples (after a total of 0, 24, and 48 h), the aforementioned parameter was calculated, which value was in the range of 0.060–0.512 MPa. Statistical analysis of the modulus of elasticity values of individual hydrogels after 48 h of incubation is presented in Fig. 9.

A non-parametric Kruskal-Wallis test was also performed. Statistically significant differences between the modulus of elasticity after 24 and 48 h can be found in the hydrogel compositions (Table 4). The significance level was assumed at *p* < 0.05.

**Table 4:** Test results from strength measurements of the prepared hydrogels. Results of Kruskal-Wallis test for statistical analyses of three types of measurements of elastic modulus of individual hydrogels for significance level *p* < 0.05. (*N* = 6)

	Hydrogel	Incubation time	0 *R*: 6.1667	24 *R*: 7.8333	48 *R*: 14.5000
H1	4% sodium alginate + 4% chitosan + DI water	0		1.0000	**0.0206**
		24	1.0000		0.0916
		48	**0.0206**	0.0916	
		Incubation time	0 *R*: 15.500	24 *R*: 5.6667	48 *R*: 7.3333
H2	4% sodium alginate + 4% gelatin + DI water	0		**0.0043**	**0.0242**
		24	**0.0043**		1.0000
		48	**0.0242**	1.0000	
		Incubation time	0 *R*: 10.333	24 *R*: 3.8333	48 *R*: 14.333
H3	4% sodium alginate + 2% agar + 2% methylocellulose + DI water	0		0.1049	0.5831
		24	0.1049		**0.0020**
		48	0.5831	**0.0020**	
		Incubation time	0 *R*: 15.500	24 *R*: 6.1667	48 *R*: 6.8333
H4	3% sodium alginate + 7% gelatin + NaCl	0		**0.0074**	**0.0148**
		24	**0.0074**		1.0000
		48	**0.0148**	1.0000	
		Incubation time	0 *R*: 7.6667	24 *R*: 5.5000	48 *R*: 15.333
H5	4% sodium alginate + 4% chitosan + NaCl	0		1.0000	**0.0386**
		24	1.0000		**0.0043**
		48	**0.0386**	**0.0043**	
		Incubation time	0 *R*: 15.500	24 *R*: 8.1667	48 *R*: 4.8333
H6	4% sodium alginate + 2% agar + 2% methylocellulose + NaCl	0		0.0520	**0.0016**
		24	0.0520		0.8385
		48	**0.0016**	0.8385	

During statistical analyses, significant differences at significance level *p* < 0.05 have been observed of the modulus of elasticity before and after incubation in the following hydrogels:

- Statistically significant differences of H1 and H6 hydrogels were observed between control group and after 48 h of incubation.
- The elastic modulus of H2 and H4 hydrogels exhibits statistically significant differences between groups after both 24 and 48 h relative to the control.
- Statistically significant difference of hydrogel H3 was noticed between group 24 h and 48 h after incubation.
- The modulus of elasticity of hydrogel H5 indicates statistically significant differences between the control group and after 48 h of incubation, as well as between 24 and 48 h after incubation.

Due to the possibility of hydrogel degradation, the elastic modulus of the hydrogel matrices varied with the duration of incubation. Polymer bonds may weaken over time, thereby affecting the material mechanical properties. The stability of the hydrogel is also affected by cell growth. The waste products of cell metabolism have a significant impact on the rate of debonding within the hydrogel structure. This degradation process creates space for new proliferating cells and improves their growth environment, which can be advantageous during cell culturing.

## Discussion

With a view to the obtained results, it can be concluded that prepared hydrogel inks exhibit appropriate performance and can be applied for 3D bio-printing of microfluidic structures utilizing commercially available bio-printer and next, for the purpose of cell culturing research. Hybrid LOC of a simple geometry was proposed to verify the technology compatibility encompassing multi-jet 3D printing and hydrogel bio-printing of the substrates with a 0.1 M CaCl_2_ cross-linking necessity. Multi-material approach was used to obtain a mechanically durable structure with entirely hydrogel-based cavity for cell inoculation and handling.

Six different hydrogel inks were composed, including different proportions of sodium alginate, agar, chitosan, gelatin, and methylcellulose. Three of the mixtures were manufactured using DI water and the other three with a saline solution (0.9% NaCl). Depending on the ink type, slightly different parameters of the 3D bio-printing process were applied covering temperature of the ink (28 °C), printhead speed (13–15 mm/s), extrusion pressure (10–12 kPa), and temperature of the table (27 °C) and head (60 °C).

In order to evaluate the mechanical properties of the structures, compression tests were conducted under natural convection and elevated temperature (37 °C) conditions — environment which is faced during the cell culturing experiments. Tests of the modulus of elasticity of the hydrogels ensured to compare the obtained values of elasticity modulus with those of natural biological tissues. Our research results were found to be similar to the modulus of elasticity values of biological tissues, such as skin: 0.060–0.850 MPa [63–66], heart muscle: 0.008–0.150 MPa, or skeletal muscle: 0.005–0.170 MPa [67–70]. Observed correspondence in mechanical properties of the produced hydrogels and biological structures indicates the real possibility of using hydrogel materials in the biomedical sector.

Interesting results were also obtained during biological validation of the hydrogel-based structures — qualitative and quantitative analyses were performed utilizing LIVE/DEAD assays and Presto blue fluorescence dye. Statistica package allowed to assess the viability of the cell cultures independently after 24 h and 48 h in hydrogel-based LOCs. Herein, all the inks promoted cell development; nevertheless, the best viability was observed for H5 hydrogel composition (3% sodium alginate + 7% gelatin + 90% NaCl), reaching circa 127.2% after 24 h and 105.4% after 48 h in comparison to control group (100%). This result may be considered as especially valuable, since similar works [14, 70] report on solely 55% and 80–90% cells viability achieved in hydrogel matrices. Thus, cytotoxicity tests confirmed appropriate biocompatibility of the structures, suggesting continuation of the research with the use of the studied lung cancer cell line (H69AR), as well as other biological samples.

## Conclusion

In the paper, a three-layer sandwiched-type LOC fabricated utilizing 3D printed biocompatible resins and hydrogel matrix of the unique composition has been shown. Six hydrogel inks of the different content were developed and all of them exhibited appropriate, repeatable performance and moderate geometry reflection during 3D bio-print. As the mechanical parameters, i.e., elasticity modulus were determined and indicated as imitating some of the biological tissues, further tests on potential hydrogel cytotoxicity were conducted. Herein, all the inks exhibited acceptable biocompatibility, resulting in substantially higher cancer cells viability values that for the control samples. The outcomes seem especially promising, since the chosen lung cancer cell line (H69AR), to the best knowledge of the authors, has never been investigated on-chip. Our future research prospects encompass further studies of 3D bio-printed hydrogel-based LOCs, towards long-term culturing of cancer cells and drug resistivity tests.

## Data Availability

The authors declare that the data supporting the findings of this study are available within the paper. Should any raw data files be needed in another format they are available from the corresponding author upon reasonable request.

## Footnotes

1 The bioprinter software used here is CAD compatible and it is possible to import.STL file with a designed model of the hydrogel structure. Another solution is ready-made models available in the printer’s user panel.
